# Correction: Development of the Italian version of the Orgasmic Perception Questionnaire (OPQ)

**DOI:** 10.1371/journal.pone.0316286

**Published:** 2024-12-19

**Authors:** Marta Panzeri, Denise Mauro, Lucia Ronconi, Ana Isabel Arcos-Romero

There is an error in affiliation 4 for author Ana Isabel Arcos-Romero. The correct affiliation 4 is: Department of Psychology, Universidad Loyola Andalucía, Seville, Spain.
