# Supplementary material for: Development of the Italian version of the Orgasmic Perception Questionnaire (OPQ)
Source: PLoS One. 2023 Oct 24;18(10):e0288850. doi: 10.1371/journal.pone.0288850 (PMC10597485; doi:10.1371/journal.pone.0288850)
Supplement: S1 File — (DOCX) [file pone.0288850.s001.docx]

**Table 1S. Factor Loadings.**

| Item | F1 | F2 | F3 | F4 | F5 |
| --- | --- | --- | --- | --- | --- |
| 29 | 0,660 |  |  |  |  |
| 16 | 0,660 |  |  |  |  |
| 48 | 0,657 |  |  |  |  |
| 45 | 0,641 |  |  |  |  |
| 58 | 0,599 |  |  |  |  |
| 46 | 0,590 |  |  |  |  |
| 26 | 0,576 |  |  |  |  |
| 47 | 0,567 |  |  |  |  |
| 35 | 0,548 |  |  |  |  |
| 25 | 0,528 |  |  |  |  |
| 54 | 0,504 |  |  |  |  |
| 37 | 0,499 |  |  |  |  |
| 30 | 0,447 |  |  |  |  |
| 27 | 0,417 |  | 0,317 |  |  |
| 34 | 0,372 |  |  |  |  |
| 63 | 0,364 |  |  |  |  |
| 41 |  |  |  |  |  |
| 51 |  |  |  |  |  |
| 33 |  | -0,857 |  |  |  |
| 39 |  | -0,819 |  |  |  |
| 10 |  | -0,801 |  |  |  |
| 2 |  | -0,785 |  |  |  |
| 55 |  | -0,784 |  |  |  |
| 6 |  | -0,734 |  |  |  |
| 59 |  | -0,731 |  |  |  |
| 12 |  | -0,650 |  |  |  |
| 43 |  | -0,517 |  |  |  |
| 14 |  | -0,515 |  |  |  |
| 9 |  | -0,470 |  |  |  |
| 19 |  | -0,421 | -0,341 |  | 0,379 |
| 20 |  | -0,355 |  |  |  |
| 61 |  | -0,328 |  |  |  |
| 38 |  |  |  |  |  |
| 36 |  |  | 0,669 |  |  |
| 50 |  |  | 0,662 |  |  |
| 15 |  |  | 0,632 |  |  |
| 57 |  |  | 0,484 |  |  |
| 40 |  |  | 0,447 |  |  |
| 56 |  |  | 0,412 | -0,308 |  |
| 22 |  |  | 0,343 |  |  |
| 42 |  |  | 0,340 |  |  |
| 28 |  |  |  |  |  |
| 52 |  |  |  | -0,821 |  |
| 1 |  |  |  | -0,762 |  |
| 44 |  |  |  | -0,638 |  |
| 24 |  |  |  | -0,591 |  |
| 7 |  |  |  | -0,502 |  |
| 23 |  |  | 0,423 | -0,478 |  |
| 3 |  |  |  | -0,428 |  |
| 31 |  |  |  | -0,391 |  |
| 53 | 0,363 |  |  | -0,373 |  |
| 4 |  |  |  | -0,364 |  |
| 5 |  |  |  |  |  |
| 13 |  |  |  |  | 0,529 |
| 18 |  |  |  |  | 0,528 |
| 8 |  |  |  |  | 0,521 |
| 21 |  |  |  |  | 0,422 |
| 60 |  |  |  |  | 0,403 |
| 49 | -0,325 |  |  |  | 0,399 |
| 11 |  |  |  |  | 0,395 |
| 62 |  |  |  |  | 0,390 |
| 17 |  |  |  |  | 0,316 |
| 32 |  |  |  |  |  |

**Table 2S. Pearson's correlation between OPQ factors in the first and second administration (N = 185).**

|  | F1 test | F2 test | F3 test | F4 test | F5 test |  |  |  |  |  |
| --- | --- | --- | --- | --- | --- | --- | --- | --- | --- | --- |
| F1 retest | .810** |  |  |  |  |  | | | | |
| F2 retest |  | .820** |  |  |  |  |  |  |  |  |
| F3 retest |  |  | .644** |  |  |  |  |  |  |  |
| F4 retest |  |  |  | .785** |  |  |  |  |  |  |
| F5 retest |  |  |  |  | .756** |  |  |  |  |  |

*Note.* *** correlation is significant at the 0.01 level (2-tailed)*

**OPQ-316: Orgasmic Perception Questionnaire (OPQ; Panzeri, 2016)**

Il seguente questionario contiene delle affermazioni riguardanti l’orgasmo. Legga ogni affermazione e indichi la frequenza con cui Le capita quanto espresso dalla frase, riferendosi esclusivamente alla fase dell'orgasmo e non a quanto accade subito prima o subito dopo. Segni con una crocetta la risposta che più si avvicina al Suo modo di pensare. Le ricordiamo che non esistono risposte giuste o sbagliate. Se recentemente non ha avuto un partner, si ricordi di segnare la casella “al momento non ho un partner sessuale”.

| 1 = Mai | 2 = Raramente | 3 = Qualche volta | 4 = Spesso | 5 = Sempre | 6 = Al momento non ho un partner sessuale | 7 = Non so/ non ricordo |
| --- | --- | --- | --- | --- | --- | --- |

| 1. Ho un aumento della sudorazione | 1 | 2 | 3 | 4 | 5 | 6 | 7 |
| --- | --- | --- | --- | --- | --- | --- | --- |
| 2. Prendo poca aria | 1 | 2 | 3 | 4 | 5 | 6 | 7 |
| 3. La fatica e il dolore muscolare per lo sforzo sono molto più sopportabili rispetto ad altri momenti | 1 | 2 | 3 | 4 | 5 | 6 | 7 |
| 4. Percepisco delle contrazioni involontarie sulle gambe | 1 | 2 | 3 | 4 | 5 | 6 | 7 |
| 5. Percepisco un aumento della salivazione | 1 | 2 | 3 | 4 | 5 | 6 | 7 |
| 6. Sento dei brividi nella zona genitale | 1 | 2 | 3 | 4 | 5 | 6 | 7 |
| 7. Mi si offusca la vista | 1 | 2 | 3 | 4 | 5 | 6 | 7 |
| 8. Provo una sensazione di irrazionalità | 1 | 2 | 3 | 4 | 5 | 6 | 7 |
| 9. Mi sento fuori di me | 1 | 2 | 3 | 4 | 5 | 6 | 7 |
| 10. Percepisco l’aumento del battito cardiaco | 1 | 2 | 3 | 4 | 5 | 6 | 7 |
| 11. È gratificante | 1 | 2 | 3 | 4 | 5 | 6 | 7 |
| 12. Sento un leggero mal di testa | 1 | 2 | 3 | 4 | 5 | 6 | 7 |
| 13. Provo una sensazione di rilassamento | 1 | 2 | 3 | 4 | 5 | 6 | 7 |
| 14. Provo un senso di potenza | 1 | 2 | 3 | 4 | 5 | 6 | 7 |
| 15. I pensieri sono focalizzati sul piacere | 1 | 2 | 3 | 4 | 5 | 6 | 7 |
| 16. Se il/la partner lo prova contemporaneamente a me è come se “si incastrassero due ingranaggi” e la sensazione è piena e completa | 1 | 2 | 3 | 4 | 5 | 6 | 7 |
| 17. Percepisco l’orgasmo in modo prevalentemente fisico, senza particolari pensieri o emozioni, eccezione fatta per la forte eccitazione | 1 | 2 | 3 | 4 | 5 | 6 | 7 |
| 18. Sento un benessere generale | 1 | 2 | 3 | 4 | 5 | 6 | 7 |
| 19. Lascio la mente libera | 1 | 2 | 3 | 4 | 5 | 6 | 7 |
| 20. Sento di voler strappare qualcosa | 1 | 2 | 3 | 4 | 5 | 6 | 7 |
| 21. Mi sento più sensibile | 1 | 2 | 3 | 4 | 5 | 6 | 7 |
| 22. Mi sembra di scalare una vetta e di raggiungere il punto più alto | 1 | 2 | 3 | 4 | 5 | 6 | 7 |
| 23. Ho la sensazione di perdere il controllo | 1 | 2 | 3 | 4 | 5 | 6 | 7 |
| 24. Spero che l’altra persona non si fermi o cambi movimento per poter prolungare il più possibile quella sensazione | 1 | 2 | 3 | 4 | 5 | 6 | 7 |
| 25. Provo amore per l’altra persona | 1 | 2 | 3 | 4 | 5 | 6 | 7 |
| 26. Mi sento accaldato | 1 | 2 | 3 | 4 | 5 | 6 | 7 |
| 27. La tensione scompare | 1 | 2 | 3 | 4 | 5 | 6 | 7 |
| 28. Provo delle sensazioni di tensione crescente sempre più piacevoli che culminano con delle scariche di piacere | 1 | 2 | 3 | 4 | 5 | 6 | 7 |
| 29. Mi rilasso | 1 | 2 | 3 | 4 | 5 | 6 | 7 |
| 30. Provo un formicolio ai genitali | 1 | 2 | 3 | 4 | 5 | 6 | 7 |
| 31. Ho bisogno che ci sia buona complicità con l’altra persona | 1 | 2 | 3 | 4 | 5 | 6 | 7 |
| 32. Mi sento dissociato | 1 | 2 | 3 | 4 | 5 | 6 | 7 |
| 33. Provo la sensazione di fare qualcosa di sporco | 1 | 2 | 3 | 4 | 5 | 6 | 7 |
| 34. È un piacere di natura egoistica | 1 | 2 | 3 | 4 | 5 | 6 | 7 |
| 35. Provo una scarica di piacere molto intensa | 1 | 2 | 3 | 4 | 5 | 6 | 7 |
| 36. Il mio corpo si contorce | 1 | 2 | 3 | 4 | 5 | 6 | 7 |
| 37. Sento un forte tremolio nei muscoli del corpo | 1 | 2 | 3 | 4 | 5 | 6 | 7 |
| 38. Provo indifferenza rispetto al contesto che sto vivendo | 1 | 2 | 3 | 4 | 5 | 6 | 7 |
| 39. A volte il piacere è talmente forte e gratificante da essere quasi intollerabile | 1 | 2 | 3 | 4 | 5 | 6 | 7 |
| 40. Mi ottunde tutti i sensi | 1 | 2 | 3 | 4 | 5 | 6 | 7 |
| 41. Mi sento euforico | 1 | 2 | 3 | 4 | 5 | 6 | 7 |
| 42. Ho paura di essere inadeguato | 1 | 2 | 3 | 4 | 5 | 6 | 7 |
| 43. Mi sento più sensuale | 1 | 2 | 3 | 4 | 5 | 6 | 7 |
| 44. Provo fastidio | 1 | 2 | 3 | 4 | 5 | 6 | 7 |
| 45. Provo una sensazione di calore | 1 | 2 | 3 | 4 | 5 | 6 | 7 |
| 46. Percepisco delle contrazioni involontarie nella zona pelvica | 1 | 2 | 3 | 4 | 5 | 6 | 7 |
| 47. Per qualche secondo mi sembra di andare in apnea | 1 | 2 | 3 | 4 | 5 | 6 | 7 |
| 48. Mi sento esplodere | 1 | 2 | 3 | 4 | 5 | 6 | 7 |
| 49. La sensazione fisica è quella di uno stress che viene sfogato | 1 | 2 | 3 | 4 | 5 | 6 | 7 |
| 50. Sento dei brividi e dei fremiti alle estremità degli arti | 1 | 2 | 3 | 4 | 5 | 6 | 7 |
| 51. Mi sento in colpa | 1 | 2 | 3 | 4 | 5 | 6 | 7 |
| 52. Mi sento realizzato | 1 | 2 | 3 | 4 | 5 | 6 | 7 |
| 53. Mi sento “alleggerito” | 1 | 2 | 3 | 4 | 5 | 6 | 7 |
| 54. Mi viene da ridere | 1 | 2 | 3 | 4 | 5 | 6 | 7 |
| 55. Provo una sensazione di paura | 1 | 2 | 3 | 4 | 5 | 6 | 7 |
| 56. Ho bisogno di sentirmi a mio agio | 1 | 2 | 3 | 4 | 5 | 6 | 7 |
| 57. Mi sento in pace col mondo | 1 | 2 | 3 | 4 | 5 | 6 | 7 |
| 58. Piango di gioia | 1 | 2 | 3 | 4 | 5 | 6 | 7 |
| 59. Lo vivo in modo molto mentale e devo essere libero di potermi lasciare andare totalmente | 1 | 2 | 3 | 4 | 5 | 6 | 7 |
| 60. Le mie percezioni sono uguali di volta in volta | 1 | 2 | 3 | 4 | 5 | 6 | 7 |
| 61. Non riesco a guardare il/la partner | 1 | 2 | 3 | 4 | 5 | 6 | 7 |
| 62. È uno sfogo della mia rabbia | 1 | 2 | 3 | 4 | 5 | 6 | 7 |
| 63. Provo una sensazione che definirei quasi “extracorporea” | 1 | 2 | 3 | 4 | 5 | 6 | 7 |
| 64. Provo un gran desiderio di baciare il/la partner | 1 | 2 | 3 | 4 | 5 | 6 | 7 |
| 65. Mi sento più legato al/a partner | 1 | 2 | 3 | 4 | 5 | 6 | 7 |
| 66. Percepisco delle pulsazioni a livello dei genitali | 1 | 2 | 3 | 4 | 5 | 6 | 7 |
| 67. Provo dolore | 1 | 2 | 3 | 4 | 5 | 6 | 7 |
| 68. Percepisco un aumento della temperatura corporea | 1 | 2 | 3 | 4 | 5 | 6 | 7 |
| 69. Sento una forte tensione di tutto il corpo | 1 | 2 | 3 | 4 | 5 | 6 | 7 |
| 70. Provo una sensazione di scosse/scariche elettriche che si propagano per il corpo | 1 | 2 | 3 | 4 | 5 | 6 | 7 |
| 71. Lo stress scompare | 1 | 2 | 3 | 4 | 5 | 6 | 7 |
| 72. Provo una contrazione muscolare involontaria | 1 | 2 | 3 | 4 | 5 | 6 | 7 |
| 73. Percepisco un aumento dei battiti nelle zone genitali | 1 | 2 | 3 | 4 | 5 | 6 | 7 |
| 74. Percepisco un tremore al labbro | 1 | 2 | 3 | 4 | 5 | 6 | 7 |
| 75. Provo un formicolio, ai limiti del piacere, che parte dalle cosce e si allunga fino ai piedi | 1 | 2 | 3 | 4 | 5 | 6 | 7 |
| 76. Mi manca l’aria | 1 | 2 | 3 | 4 | 5 | 6 | 7 |
| 77. Mi sento affaticato | 1 | 2 | 3 | 4 | 5 | 6 | 7 |
| 78. Involontariamente inarco la schiena | 1 | 2 | 3 | 4 | 5 | 6 | 7 |
| 79. Il/la partner mi fa sentire unico | 1 | 2 | 3 | 4 | 5 | 6 | 7 |
| 80. La sensazione generale è che il mio corpo si estranei dalle sensazioni comuni “terrene” e che si elevi assieme a quello dell’altra persona a uno stadio più alto | 1 | 2 | 3 | 4 | 5 | 6 | 7 |
| 81. Provo uno stato di confusione assoluta | 1 | 2 | 3 | 4 | 5 | 6 | 7 |
| 82. Provo vergogna | 1 | 2 | 3 | 4 | 5 | 6 | 7 |
| 83. Mi domando se sono riuscito a soddisfare l’altra persona | 1 | 2 | 3 | 4 | 5 | 6 | 7 |
| 84. Riesco a raggiungerlo con facilità | 1 | 2 | 3 | 4 | 5 | 6 | 7 |
| 85. Ho l’impressione che sia stato meglio della volta precedente | 1 | 2 | 3 | 4 | 5 | 6 | 7 |
| 86. Ho bisogno di sentirmi rispettato dal/la partner | 1 | 2 | 3 | 4 | 5 | 6 | 7 |
| 87. Fermo il/la partner perché la sensazione che provo è così forte da non riuscire a gestirla | 1 | 2 | 3 | 4 | 5 | 6 | 7 |
| 88. Non so quello che dico o quello che faccio | 1 | 2 | 3 | 4 | 5 | 6 | 7 |
| 89. Mi sento in un limbo tra eccessiva gioia ed eccessivo dolore | 1 | 2 | 3 | 4 | 5 | 6 | 7 |
| 90. Il piacere che provo risulta troppo forte da sopportare | 1 | 2 | 3 | 4 | 5 | 6 | 7 |
| 91. Provo solo un piacere fisico (nulla di mentale) | 1 | 2 | 3 | 4 | 5 | 6 | 7 |
| 92. Chiudendo gli occhi vedo immagini ogni volta diverse | 1 | 2 | 3 | 4 | 5 | 6 | 7 |
| 93. Ho la sensazione di svenire | 1 | 2 | 3 | 4 | 5 | 6 | 7 |
| 94. Provo una sensazione di onnipotenza sul/la partner | 1 | 2 | 3 | 4 | 5 | 6 | 7 |
| 95. Mi sento leggero | 1 | 2 | 3 | 4 | 5 | 6 | 7 |
| 96. Mi sembra di non avere un controllo sulla situazione | 1 | 2 | 3 | 4 | 5 | 6 | 7 |
| 97. Mi sento triste | 1 | 2 | 3 | 4 | 5 | 6 | 7 |
| 98. Percepisco un piacere fisico localizzato a livello genitale | 1 | 2 | 3 | 4 | 5 | 6 | 7 |
| 99. Provo un fremito lungo tutto il corpo | 1 | 2 | 3 | 4 | 5 | 6 | 7 |
| 100. La scossa elettrica viene a gradi, da piano diventa sempre più forte | 1 | 2 | 3 | 4 | 5 | 6 | 7 |
| 101. Sento calore sul viso | 1 | 2 | 3 | 4 | 5 | 6 | 7 |
| 102. Sento delle contrazioni muscolari che si diffondono dall’area genitale al resto del corpo | 1 | 2 | 3 | 4 | 5 | 6 | 7 |
| 103. Sento i capezzoli che si irrigidiscono | 1 | 2 | 3 | 4 | 5 | 6 | 7 |
| 104. Il respiro si fa irregolare | 1 | 2 | 3 | 4 | 5 | 6 | 7 |
| 105. Mi sento pervaso da una scarica di energia | 1 | 2 | 3 | 4 | 5 | 6 | 7 |
| 106. Percepisco un’accelerazione del respiro | 1 | 2 | 3 | 4 | 5 | 6 | 7 |
| 107. Mi sento completamente rilassato a livello muscolare | 1 | 2 | 3 | 4 | 5 | 6 | 7 |
| 108. Provo un senso di abbandono generale | 1 | 2 | 3 | 4 | 5 | 6 | 7 |
| 109. Rifletto su cosa potrebbero pensare le altre persone se sapessero cosa faccio | 1 | 2 | 3 | 4 | 5 | 6 | 7 |
| 110. Sento delle ondate di piacere che arrivano a un picco massimo | 1 | 2 | 3 | 4 | 5 | 6 | 7 |
| 111. Le emozioni che provo sono tutte positive | 1 | 2 | 3 | 4 | 5 | 6 | 7 |
| 112. Mi sento estasiato | 1 | 2 | 3 | 4 | 5 | 6 | 7 |
| 113. Mi sento sporco | 1 | 2 | 3 | 4 | 5 | 6 | 7 |
| 114. Sento di essere andato contro me stesso (per accontentare l’altra persona) | 1 | 2 | 3 | 4 | 5 | 6 | 7 |
| 115. Non è facile lasciarsi andare | 1 | 2 | 3 | 4 | 5 | 6 | 7 |
| 116. Ho una sensazione di onnipotenza dovuta a una sensazione di soddisfazione totale | 1 | 2 | 3 | 4 | 5 | 6 | 7 |
| 117. Sento una completa incapacità di pensare ad altro | 1 | 2 | 3 | 4 | 5 | 6 | 7 |
| 118. Mi concentro su me stesso e sul/la partner, il resto non esiste | 1 | 2 | 3 | 4 | 5 | 6 | 7 |
| 119. Riesco a raggiungerlo con fatica | 1 | 2 | 3 | 4 | 5 | 6 | 7 |
| 120. Ho voglia di urlare | 1 | 2 | 3 | 4 | 5 | 6 | 7 |
| 121. Provo delle sensazioni contrastanti: da un lato voglio provarlo dall’altro tento di posticiparlo perché è un’esperienza che dura un istante | 1 | 2 | 3 | 4 | 5 | 6 | 7 |
| 122. Si accavallano delle immagini mentali che non riesco a distinguere | 1 | 2 | 3 | 4 | 5 | 6 | 7 |
| 123. Non riesco a sentire il mio corpo | 1 | 2 | 3 | 4 | 5 | 6 | 7 |
| 124. Provo un senso di rabbia | 1 | 2 | 3 | 4 | 5 | 6 | 7 |
| 125. Provo una sensazione di leggerezza | 1 | 2 | 3 | 4 | 5 | 6 | 7 |
| 126. Mi sento rigenerato | 1 | 2 | 3 | 4 | 5 | 6 | 7 |
| 127. Provo un brivido che anestetizza la zona genitale | 1 | 2 | 3 | 4 | 5 | 6 | 7 |
| 128. Provo una sensazione di calore nella zona genitale | 1 | 2 | 3 | 4 | 5 | 6 | 7 |
| 129. Percepisco tensione muscolare | 1 | 2 | 3 | 4 | 5 | 6 | 7 |
| 130. Vengo investito da un’ondata di piacere che parte dalla zona genitale e si irradia fino alla testa | 1 | 2 | 3 | 4 | 5 | 6 | 7 |
| 131. Percepisco delle contrazioni involontarie sui fianchi | 1 | 2 | 3 | 4 | 5 | 6 | 7 |
| 132. Ho il respiro affannoso | 1 | 2 | 3 | 4 | 5 | 6 | 7 |
| 133. Sento dei brividi lungo le gambe sino ai piedi | 1 | 2 | 3 | 4 | 5 | 6 | 7 |
| 134. Mi sembra un sogno | 1 | 2 | 3 | 4 | 5 | 6 | 7 |
| 135. Provo solo un piacere mentale (nulla di fisico) | 1 | 2 | 3 | 4 | 5 | 6 | 7 |
| 136. Mi preoccupo per lo stato psicofisico dell’altra persona | 1 | 2 | 3 | 4 | 5 | 6 | 7 |
| 137. Si susseguono in me pensieri positivi riguardo alla realtà | 1 | 2 | 3 | 4 | 5 | 6 | 7 |
| 138. Mi sento ricco di energia | 1 | 2 | 3 | 4 | 5 | 6 | 7 |
| 139. Sento calore a livello dell’addome | 1 | 2 | 3 | 4 | 5 | 6 | 7 |
| 140. Provo delle emozioni negative | 1 | 2 | 3 | 4 | 5 | 6 | 7 |
| 141. Provo una sensazione di liberazione | 1 | 2 | 3 | 4 | 5 | 6 | 7 |
| 142. Associo all’orgasmo un’immagine nella mente | 1 | 2 | 3 | 4 | 5 | 6 | 7 |
| 143. Se durasse di più non so se riuscirei a sopportarlo | 1 | 2 | 3 | 4 | 5 | 6 | 7 |
| 144. Vorrei durasse sempre di più | 1 | 2 | 3 | 4 | 5 | 6 | 7 |
| 145. Mi abbandono alle sensazioni escludendo il mondo esterno | 1 | 2 | 3 | 4 | 5 | 6 | 7 |
| 146. Non compaiono pensieri articolati | 1 | 2 | 3 | 4 | 5 | 6 | 7 |
| 147. Mi lacrimano gli occhi | 1 | 2 | 3 | 4 | 5 | 6 | 7 |
| 148. Provo una sensazione di pace con me stesso | 1 | 2 | 3 | 4 | 5 | 6 | 7 |
| 149. Sento di voler stringere forte a me il/la partner | 1 | 2 | 3 | 4 | 5 | 6 | 7 |
| 150. È come se nel mio corpo si accumulasse energia che si rilascia in ondate sempre maggiori | 1 | 2 | 3 | 4 | 5 | 6 | 7 |
| 151. Provo gratitudine per il/la partner | 1 | 2 | 3 | 4 | 5 | 6 | 7 |
| 152. Ho la sensazione di essere isolato dal resto del mondo | 1 | 2 | 3 | 4 | 5 | 6 | 7 |
| 153. L’orgasmo del/la partner influenza in modo positivo il mio orgasmo | 1 | 2 | 3 | 4 | 5 | 6 | 7 |
| 154. Ho difficoltà a distinguere le emozioni | 1 | 2 | 3 | 4 | 5 | 6 | 7 |
| 155. Mi sento lontano da ogni cosa, in un’altra dimensione, una realtà parallela | 1 | 2 | 3 | 4 | 5 | 6 | 7 |
| 156. Provo quasi paura a lasciarmi andare | 1 | 2 | 3 | 4 | 5 | 6 | 7 |
| 157. Ho paura di star venendo troppo presto | 1 | 2 | 3 | 4 | 5 | 6 | 7 |
| 158. Ho l’impressione che i pensieri si accavallino per poi sfogarsi nel pianto | 1 | 2 | 3 | 4 | 5 | 6 | 7 |
| 159. Se ho dei pensieri, poi non li ricordo | 1 | 2 | 3 | 4 | 5 | 6 | 7 |
| 160. Le immagini mentali che provo in questo momento non le ricordo più | 1 | 2 | 3 | 4 | 5 | 6 | 7 |
| 161. Mi sembra che intorno tutto sia perfetto | 1 | 2 | 3 | 4 | 5 | 6 | 7 |
| 162. Provo una forte sensazione di volerne sempre di più | 1 | 2 | 3 | 4 | 5 | 6 | 7 |
| 163. Mi sento come in paradiso | 1 | 2 | 3 | 4 | 5 | 6 | 7 |
| 164. Sento la voglia di lasciarmi andare completamente, senza avere alcuna inibizione rispetto al mio corpo e alla mia fisicità | 1 | 2 | 3 | 4 | 5 | 6 | 7 |
| 165. Provo una gioia infinita | 1 | 2 | 3 | 4 | 5 | 6 | 7 |
| 166. Mi succede di provare fastidio e di non riuscire a vivere del tutto il momento, come se provassi vergogna | 1 | 2 | 3 | 4 | 5 | 6 | 7 |
| 167. Perdo il senso del mio confine corporeo | 1 | 2 | 3 | 4 | 5 | 6 | 7 |
| 168. Sento una forte scarica, simile ad uno spasmo che si colloca a metà tra piacere e dolore | 1 | 2 | 3 | 4 | 5 | 6 | 7 |
| 169. Se l’altro/a non prova l’orgasmo insieme a me la sensazione è meno bella | 1 | 2 | 3 | 4 | 5 | 6 | 7 |
| 170. Ho il cuore a mille | 1 | 2 | 3 | 4 | 5 | 6 | 7 |
| 171. La mia coscienza è assente e disinteressata | 1 | 2 | 3 | 4 | 5 | 6 | 7 |
| 172. Mi sento più realizzato quando sono io a dare piacere al/la partner | 1 | 2 | 3 | 4 | 5 | 6 | 7 |
| 173. Scompare qualsiasi ansia | 1 | 2 | 3 | 4 | 5 | 6 | 7 |
| 174. Non ho alcun tipo di inibizione, di freno o di limite | 1 | 2 | 3 | 4 | 5 | 6 | 7 |
| 175. Mi sento determinato a raggiungerlo a qualunque costo | 1 | 2 | 3 | 4 | 5 | 6 | 7 |
| 176. I momenti stressanti influiscono negativamente sulla percezione dell’orgasmo | 1 | 2 | 3 | 4 | 5 | 6 | 7 |
| 177. Non mi preoccupo di far raggiungere l’orgasmo anche al/la partner | 1 | 2 | 3 | 4 | 5 | 6 | 7 |
| 178. Sento vibrare il corpo come in una scarica elettrica | 1 | 2 | 3 | 4 | 5 | 6 | 7 |
| 179. Provo contrazione e rilassamento corporeo piuttosto rapidi e intensi | 1 | 2 | 3 | 4 | 5 | 6 | 7 |
| 180. Mi sento intorpidito | 1 | 2 | 3 | 4 | 5 | 6 | 7 |
| 181. In testa compaiono luci e colori | 1 | 2 | 3 | 4 | 5 | 6 | 7 |
| 182. Ho la sensazione che la parte più istintiva di me prenda il sopravvento | 1 | 2 | 3 | 4 | 5 | 6 | 7 |
| 183. Provo una sensazione inebriante che offusca i miei pensieri | 1 | 2 | 3 | 4 | 5 | 6 | 7 |
| 184. Provo una sensazione di malinconia | 1 | 2 | 3 | 4 | 5 | 6 | 7 |
| 185. Provo estremo piacere | 1 | 2 | 3 | 4 | 5 | 6 | 7 |
| 186. Mi sento per un attimo fuori dalla realtà | 1 | 2 | 3 | 4 | 5 | 6 | 7 |
| 187. Mi manca il respiro | 1 | 2 | 3 | 4 | 5 | 6 | 7 |
| 188. Mi sento immerso in una totale follia | 1 | 2 | 3 | 4 | 5 | 6 | 7 |
| 189. Provo una sensazione di tranquillità | 1 | 2 | 3 | 4 | 5 | 6 | 7 |
| 190. Provo un senso di onnipotenza | 1 | 2 | 3 | 4 | 5 | 6 | 7 |
| 191. Sento il desiderio che il/la partner provi quello che sto provando io | 1 | 2 | 3 | 4 | 5 | 6 | 7 |
| 192. Sento dei brividi lungo le gambe | 1 | 2 | 3 | 4 | 5 | 6 | 7 |
| 193. Provo un formicolio a mani e piedi | 1 | 2 | 3 | 4 | 5 | 6 | 7 |
| 194. A volte è così gradevole che ho l’impressione che sia “troppo” | 1 | 2 | 3 | 4 | 5 | 6 | 7 |
| 195. Provo un senso di liberazione psicologica | 1 | 2 | 3 | 4 | 5 | 6 | 7 |
| 196. Ho la sensazione che il tempo si sia fermato | 1 | 2 | 3 | 4 | 5 | 6 | 7 |
| 197. Sento di amare profondamente colui/lei che mi ha provocato questo piacere | 1 | 2 | 3 | 4 | 5 | 6 | 7 |
| 198. È fisicamente paralizzante | 1 | 2 | 3 | 4 | 5 | 6 | 7 |
| 199. È come se le tensioni fluissero dopo “aver fatto saltare un resistente tappo” | 1 | 2 | 3 | 4 | 5 | 6 | 7 |
| 200. Traggo piacere dall’orgasmo del/la partner | 1 | 2 | 3 | 4 | 5 | 6 | 7 |
| 201. Sento una forte scossa elettrica che mi attraversa | 1 | 2 | 3 | 4 | 5 | 6 | 7 |
| 202. Sento una sorta di brivido | 1 | 2 | 3 | 4 | 5 | 6 | 7 |
| 203. Avverto la contrazione dei muscoli | 1 | 2 | 3 | 4 | 5 | 6 | 7 |
| 204. Sento una breve scarica che finisce con lo stancarmi molto | 1 | 2 | 3 | 4 | 5 | 6 | 7 |
| 205. La mente si libera da altri pensieri | 1 | 2 | 3 | 4 | 5 | 6 | 7 |
| 206. Ho bisogno di sentirmi rilassato | 1 | 2 | 3 | 4 | 5 | 6 | 7 |
| 207. Sono imbarazzato | 1 | 2 | 3 | 4 | 5 | 6 | 7 |
| 208. Ho paura di stare deludendo il/la partner | 1 | 2 | 3 | 4 | 5 | 6 | 7 |
| 209. E’ un’emozione estrema | 1 | 2 | 3 | 4 | 5 | 6 | 7 |
| 210. A volte non capisco da dove arrivi | 1 | 2 | 3 | 4 | 5 | 6 | 7 |
| 211. Mi sento vulnerabile | 1 | 2 | 3 | 4 | 5 | 6 | 7 |
| 212. Sento un’amplificazione di tutte le emozioni e sensazioni, sia fisiche che mentali | 1 | 2 | 3 | 4 | 5 | 6 | 7 |
| 213. Mi sento più disinibito | 1 | 2 | 3 | 4 | 5 | 6 | 7 |
| 214. Ho una sensazione di estrema comunione con tutto ciò che ho intorno | 1 | 2 | 3 | 4 | 5 | 6 | 7 |
| 215. Mi sento protetto dal/la partner | 1 | 2 | 3 | 4 | 5 | 6 | 7 |
| 216. Ho l’impressione che ci sia un moto vorticoso nelle viscere | 1 | 2 | 3 | 4 | 5 | 6 | 7 |
| 217. Sento i muscoli delle gambe irrigidirsi | 1 | 2 | 3 | 4 | 5 | 6 | 7 |
| 218. Ho la sensazione di diventare un tutt’uno con l’altra persona | 1 | 2 | 3 | 4 | 5 | 6 | 7 |
| 219. Mi sento in colpa per il piacere raggiunto | 1 | 2 | 3 | 4 | 5 | 6 | 7 |
| 220. Mi sento totalmente egocentrico, vorrei che l’altra persona sparisse | 1 | 2 | 3 | 4 | 5 | 6 | 7 |
| 221. Provo un “senso di vittoria” | 1 | 2 | 3 | 4 | 5 | 6 | 7 |
| 222. Lo vivo come un qualcosa di angosciante | 1 | 2 | 3 | 4 | 5 | 6 | 7 |
| 223. Mi sento come se non avessi nessuna preoccupazione | 1 | 2 | 3 | 4 | 5 | 6 | 7 |
| 224. Provo una scarica di energia seguita da rilassamento | 1 | 2 | 3 | 4 | 5 | 6 | 7 |
| 225. Penso che non vorrei essere lì in quel momento | 1 | 2 | 3 | 4 | 5 | 6 | 7 |
| 226. L’intensità dei miei orgasmi è abbastanza variabile | 1 | 2 | 3 | 4 | 5 | 6 | 7 |
| 227. Non sento di essere andato contro me stesso (per accontentare l’altra persona) | 1 | 2 | 3 | 4 | 5 | 6 | 7 |
| 228. Mi sento sereno | 1 | 2 | 3 | 4 | 5 | 6 | 7 |
| 229. Ho l’impressione di smettere di sentire i muscoli | 1 | 2 | 3 | 4 | 5 | 6 | 7 |
| 230. Mi preoccupo di far raggiungere l’orgasmo anche al/la partner | 1 | 2 | 3 | 4 | 5 | 6 | 7 |
| 231. Sento come se il corpo si allungasse fino a dividersi in due parti | 1 | 2 | 3 | 4 | 5 | 6 | 7 |
| 232. Percepisco delle contrazioni involontarie sui piedi | 1 | 2 | 3 | 4 | 5 | 6 | 7 |
| 233. Sento l’esigenza di divaricare le gambe | 1 | 2 | 3 | 4 | 5 | 6 | 7 |
| 234. Sono concentrato sul/la partner | 1 | 2 | 3 | 4 | 5 | 6 | 7 |
| 235. Mi sento confuso | 1 | 2 | 3 | 4 | 5 | 6 | 7 |
| 236. La mia parte razionale si annulla | 1 | 2 | 3 | 4 | 5 | 6 | 7 |
| 237. La mia mente è occupata solo dal piacere e dall’oggetto sessuale che lo stimola | 1 | 2 | 3 | 4 | 5 | 6 | 7 |
| 238. Ho voglia di dormire e allo stesso tempo di continuare | 1 | 2 | 3 | 4 | 5 | 6 | 7 |
| 239. Ho la sensazione che il corpo “si muova da sé” | 1 | 2 | 3 | 4 | 5 | 6 | 7 |
| 240. Mi sento più coinvolto se nello stesso momento prova l’orgasmo anche l’altra persona | 1 | 2 | 3 | 4 | 5 | 6 | 7 |
| 241. Sento che la mia percezione della realtà è falsata | 1 | 2 | 3 | 4 | 5 | 6 | 7 |
| 242. Mi tremano le gambe | 1 | 2 | 3 | 4 | 5 | 6 | 7 |
| 243. Sento dei brividi a livello del bacino | 1 | 2 | 3 | 4 | 5 | 6 | 7 |
| 244. Ho la sensazione di essere solo ed è l’unica cosa importante in quel momento | 1 | 2 | 3 | 4 | 5 | 6 | 7 |
| 245. Provo un forte contatto emotivo con l’altra persona | 1 | 2 | 3 | 4 | 5 | 6 | 7 |
| 246. Sento una totalità tra mente e corpo | 1 | 2 | 3 | 4 | 5 | 6 | 7 |
| 247. È come se la mia mente si spegnesse | 1 | 2 | 3 | 4 | 5 | 6 | 7 |
| 248. Non penso a nulla | 1 | 2 | 3 | 4 | 5 | 6 | 7 |
| 249. Mi sento insicuro | 1 | 2 | 3 | 4 | 5 | 6 | 7 |
| 250. Provo una sensazione di estremo benessere | 1 | 2 | 3 | 4 | 5 | 6 | 7 |
| 251. Mi preoccupo di raggiungere io l’orgasmo | 1 | 2 | 3 | 4 | 5 | 6 | 7 |
| 252. È come se la realtà si discostasse da me | 1 | 2 | 3 | 4 | 5 | 6 | 7 |
| 253. Provo un senso di vuoto | 1 | 2 | 3 | 4 | 5 | 6 | 7 |
| 254. I pensieri che provo in questo momento possono variare di volta in volta | 1 | 2 | 3 | 4 | 5 | 6 | 7 |
| 255. Provo calma | 1 | 2 | 3 | 4 | 5 | 6 | 7 |
| 256. Mi sento realizzato | 1 | 2 | 3 | 4 | 5 | 6 | 7 |
| 257. L’orgasmo del/la partner influenza in modo negativo il mio orgasmo | 1 | 2 | 3 | 4 | 5 | 6 | 7 |
| 258. Provo un senso di completa comunione con l’altra persona | 1 | 2 | 3 | 4 | 5 | 6 | 7 |
| 259. Percepisco delle contrazioni involontarie sulla schiena | 1 | 2 | 3 | 4 | 5 | 6 | 7 |
| 260. Provo dolore nella zona genitale | 1 | 2 | 3 | 4 | 5 | 6 | 7 |
| 261. Sento che i miei sensi sono amplificati | 1 | 2 | 3 | 4 | 5 | 6 | 7 |
| 262. Mi sento forte | 1 | 2 | 3 | 4 | 5 | 6 | 7 |
| 263. Provo un piacere quasi trascendentale | 1 | 2 | 3 | 4 | 5 | 6 | 7 |
| 264. Ho la sensazione che la realtà intorno sia banale | 1 | 2 | 3 | 4 | 5 | 6 | 7 |
| 265. Sento una sorta di rigidità muscolare | 1 | 2 | 3 | 4 | 5 | 6 | 7 |
| 266. Mi sento parte dell’altra persona | 1 | 2 | 3 | 4 | 5 | 6 | 7 |
| 267. E’ un qualcosa di travolgente | 1 | 2 | 3 | 4 | 5 | 6 | 7 |
| 268. I pensieri accelerano fino ad essere indistinguibili | 1 | 2 | 3 | 4 | 5 | 6 | 7 |
| 269. Ho la sensazione di libertà assoluta | 1 | 2 | 3 | 4 | 5 | 6 | 7 |
| 270. Provo uno spasmo addominale | 1 | 2 | 3 | 4 | 5 | 6 | 7 |
| 271. Sento dei brividi lungo la schiena | 1 | 2 | 3 | 4 | 5 | 6 | 7 |
| 272. Sento bisogno dell’altra persona | 1 | 2 | 3 | 4 | 5 | 6 | 7 |
| 273. Mi sento in estasi | 1 | 2 | 3 | 4 | 5 | 6 | 7 |
| 274. Provo emozioni molto forti e positive | 1 | 2 | 3 | 4 | 5 | 6 | 7 |
| 275. Mi sento vuoto | 1 | 2 | 3 | 4 | 5 | 6 | 7 |
| 276. Mi concentro totalmente sul mio corpo | 1 | 2 | 3 | 4 | 5 | 6 | 7 |
| 277. Mi concentro esclusivamente sulle sensazioni fisiche e sulle emozioni | 1 | 2 | 3 | 4 | 5 | 6 | 7 |
| 278. Non riesco a rimanere in silenzio | 1 | 2 | 3 | 4 | 5 | 6 | 7 |
| 279. Sento dei tremori | 1 | 2 | 3 | 4 | 5 | 6 | 7 |
| 280. Percepisco una sensazione di “solletico” nella zona genitale | 1 | 2 | 3 | 4 | 5 | 6 | 7 |
| 281. Provo un senso di perdita del controllo di emozioni, pensieri e azioni | 1 | 2 | 3 | 4 | 5 | 6 | 7 |
| 282. Provo una sensazione di conforto | 1 | 2 | 3 | 4 | 5 | 6 | 7 |
| 283. Ho degli spasmi involontari | 1 | 2 | 3 | 4 | 5 | 6 | 7 |
| 284. Percepisco una forza concentrata sulla pancia | 1 | 2 | 3 | 4 | 5 | 6 | 7 |
| 285. Mi sento egocentrico | 1 | 2 | 3 | 4 | 5 | 6 | 7 |
| 286. Mi viene da sorridere | 1 | 2 | 3 | 4 | 5 | 6 | 7 |
| 287. Spero finisca presto | 1 | 2 | 3 | 4 | 5 | 6 | 7 |
| 288. In quel preciso momento è puro istinto, senza lasciare molto spazio ai pensieri | 1 | 2 | 3 | 4 | 5 | 6 | 7 |
| 289. È come se il corpo si annullasse | 1 | 2 | 3 | 4 | 5 | 6 | 7 |
| 290. Mi sento inadeguato | 1 | 2 | 3 | 4 | 5 | 6 | 7 |
| 291. Sento che il mio corpo è attraversato da spasmi | 1 | 2 | 3 | 4 | 5 | 6 | 7 |
| 292. Mi sento molto più in relazione con l’altra persona | 1 | 2 | 3 | 4 | 5 | 6 | 7 |
| 293. Mi sento felice | 1 | 2 | 3 | 4 | 5 | 6 | 7 |
| 294. Mi sento agitato | 1 | 2 | 3 | 4 | 5 | 6 | 7 |
| 295. Mi sento etereo | 1 | 2 | 3 | 4 | 5 | 6 | 7 |
| 296. Provo un’emozione così forte che sento di dover piangere | 1 | 2 | 3 | 4 | 5 | 6 | 7 |
| 297. Percepisco delle contrazioni involontarie sull’addome | 1 | 2 | 3 | 4 | 5 | 6 | 7 |
| 298. Sento una sensazione di pienezza, abbondanza | 1 | 2 | 3 | 4 | 5 | 6 | 7 |
| 299. Ho paura di non andar bene | 1 | 2 | 3 | 4 | 5 | 6 | 7 |
| 300. Non riesco a distinguere bene i confini tra me e il resto | 1 | 2 | 3 | 4 | 5 | 6 | 7 |
| 301. E’ una sensazione forte che fa sentire tutti i pensieri | 1 | 2 | 3 | 4 | 5 | 6 | 7 |
| 302. Ho voglia di mordere qualcosa | 1 | 2 | 3 | 4 | 5 | 6 | 7 |
| 303. Mi focalizzo su quello che succede | 1 | 2 | 3 | 4 | 5 | 6 | 7 |
| 304. Non riesco a distinguere le sensazioni | 1 | 2 | 3 | 4 | 5 | 6 | 7 |
| 305. Ho la pelle d’oca | 1 | 2 | 3 | 4 | 5 | 6 | 7 |
| 306. Provo una leggera ansia | 1 | 2 | 3 | 4 | 5 | 6 | 7 |
| 307. Piango perché sono triste | 1 | 2 | 3 | 4 | 5 | 6 | 7 |
| 308. Provo esperienze di orgasmo diverse tra loro | 1 | 2 | 3 | 4 | 5 | 6 | 7 |
| 309. Ho paura di essere giudicato | 1 | 2 | 3 | 4 | 5 | 6 | 7 |
| 310. Mi sento bene | 1 | 2 | 3 | 4 | 5 | 6 | 7 |
| 311. Non mi concentro su nulla in particolare | 1 | 2 | 3 | 4 | 5 | 6 | 7 |
| 312. Mi sento appagato | 1 | 2 | 3 | 4 | 5 | 6 | 7 |
| 313. Ho paura che la mia prestazione sia inadeguata | 1 | 2 | 3 | 4 | 5 | 6 | 7 |
| 314. Non posso essere distratto | 1 | 2 | 3 | 4 | 5 | 6 | 7 |
| 315. Provo una soddisfazione totale | 1 | 2 | 3 | 4 | 5 | 6 | 7 |
| 316. Sento il bisogno di stringere qualcosa | 1 | 2 | 3 | 4 | 5 | 6 | 7 |

The underlined items are different in the masculine and feminine versions.

**OPQ-63: Orgasmic Perception Questionnaire (OPQ; Panzeri, 2016)**

Il seguente questionario contiene delle affermazioni riguardanti l’orgasmo. Legga ogni affermazione e indichi la frequenza con cui Le capita quanto espresso dalla frase, riferendosi esclusivamente alla fase dell'orgasmo e non a quanto accade subito prima o subito dopo. L’orgasmo può essere provato in diversi modi: Le chiediamo di rispondere sulla base di quello che Lei prova solitamente, riferendosi soprattutto agli ultimi orgasmi provati. Segni con una crocetta la risposta che più si avvicina al Suo modo di pensare e alle sensazioni da Lei provate o, nel caso in cui non abbia mai provato l’orgasmo, si immagini come sarebbe provare un orgasmo. Le ricordiamo che non esistono risposte giuste o sbagliate. Se non ha mai avuto un/a partner, barri pure la casella “non ho mai avuto un/a partner” nelle frasi in cui ci si riferisce esplicitamente ad esso/a.

Nel caso in cui **non avesse sperimentato un orgasmo**, La preghiamo di leggere quanto segue e di rispondere alle frasi del questionario immaginandosi come sarebbe provare un orgasmo.

| 1 = Mai | 2 = Raramente | 3 = Qualche volta | 4 = Spesso | 5 = Sempre | 6 = Non ho mai avuto un/a partner sessuale | 7 = Non so/ non ricordo |
| --- | --- | --- | --- | --- | --- | --- |

| 1. Provo un senso di potenza | 1 | 2 | 3 | 4 | 5 | 6 | 7 |
| --- | --- | --- | --- | --- | --- | --- | --- |
| 2. Sento un forte tremolio nei muscoli del corpo | 1 | 2 | 3 | 4 | 5 | 6 | 7 |
| 3. Mi sento euforico | 1 | 2 | 3 | 4 | 5 | 6 | 7 |
| 4. Mi sento più sensuale | 1 | 2 | 3 | 4 | 5 | 6 | 7 |
| 5. La sensazione fisica è quella di uno stress che viene sfogato | 1 | 2 | 3 | 4 | 5 | 6 | 7 |
| 6. Sento dei brividi e dei fremiti alle estremità degli arti | 1 | 2 | 3 | 4 | 5 | 6 | 7 |
| 7. Mi sento realizzato | 1 | 2 | 3 | 4 | 5 | 6 | 7 |
| 8. Percepisco un aumento della temperatura corporea | 1 | 2 | 3 | 4 | 5 | 6 | 7 |
| 9. Sento una forte tensione di tutto il corpo | 1 | 2 | 3 | 4 | 5 | 6 | 7 |
| 10. Provo una sensazione di scosse/scariche elettriche che si propagano per il corpo | 1 | 2 | 3 | 4 | 5 | 6 | 7 |
| 11. Percepisco un piacere fisico localizzato a livello genitale | 1 | 2 | 3 | 4 | 5 | 6 | 7 |
| 12. Provo un fremito lungo tutto il corpo | 1 | 2 | 3 | 4 | 5 | 6 | 7 |
| 13. Sento calore sul viso | 1 | 2 | 3 | 4 | 5 | 6 | 7 |
| 14. Sento delle contrazioni muscolari che si diffondono dall’area genitale al resto del corpo | 1 | 2 | 3 | 4 | 5 | 6 | 7 |
| 15. Mi sento completamente rilassato a livello muscolare | 1 | 2 | 3 | 4 | 5 | 6 | 7 |
| 16. Sento una completa incapacità di pensare ad altro | 1 | 2 | 3 | 4 | 5 | 6 | 7 |
| 17. Ho voglia di urlare | 1 | 2 | 3 | 4 | 5 | 6 | 7 |
| 18. Provo una sensazione di calore nella zona genitale | 1 | 2 | 3 | 4 | 5 | 6 | 7 |
| 19. Percepisco tensione muscolare | 1 | 2 | 3 | 4 | 5 | 6 | 7 |
| 20. Vengo investito da un’ondata di piacere che parte dalla zona genitale e si irradia fino alla testa | 1 | 2 | 3 | 4 | 5 | 6 | 7 |
| 21. Ho il respiro affannoso | 1 | 2 | 3 | 4 | 5 | 6 | 7 |
| 22. Mi preoccupo per lo stato psicofisico dell’altra persona | 1 | 2 | 3 | 4 | 5 | 6 | 7 |
| 23. Si susseguono in me pensieri positivi riguardo alla realtà | 1 | 2 | 3 | 4 | 5 | 6 | 7 |
| 24. Mi sento ricco di energia | 1 | 2 | 3 | 4 | 5 | 6 | 7 |
| 25. Non compaiono pensieri articolati | 1 | 2 | 3 | 4 | 5 | 6 | 7 |
| 26. Ho la sensazione di essere isolato dal resto del mondo | 1 | 2 | 3 | 4 | 5 | 6 | 7 |
| 27. Mi sembra che intorno tutto sia perfetto | 1 | 2 | 3 | 4 | 5 | 6 | 7 |
| 28. Se l’altro/a non prova l’orgasmo insieme a me la sensazione è meno bella | 1 | 2 | 3 | 4 | 5 | 6 | 7 |
| 29. Scompare qualsiasi ansia | 1 | 2 | 3 | 4 | 5 | 6 | 7 |
| 30. Non ho alcun tipo di inibizione, di freno o di limite | 1 | 2 | 3 | 4 | 5 | 6 | 7 |
| 31. Mi sento determinato a raggiungerlo a qualunque costo | 1 | 2 | 3 | 4 | 5 | 6 | 7 |
| 32. I momenti stressanti influiscono negativamente sulla percezione dell’orgasmo | 1 | 2 | 3 | 4 | 5 | 6 | 7 |
| 33. Sento vibrare il corpo come in una scarica elettrica | 1 | 2 | 3 | 4 | 5 | 6 | 7 |
| 34. Ho la sensazione che la parte più istintiva di me prenda il sopravvento | 1 | 2 | 3 | 4 | 5 | 6 | 7 |
| 35. Mi sento per un attimo fuori dalla realtà | 1 | 2 | 3 | 4 | 5 | 6 | 7 |
| 36. Provo una sensazione di tranquillità | 1 | 2 | 3 | 4 | 5 | 6 | 7 |
| 37. Ho la sensazione che il tempo si sia fermato | 1 | 2 | 3 | 4 | 5 | 6 | 7 |
| 38. È come se le tensioni fluissero dopo “aver fatto saltare un resistente tappo” | 1 | 2 | 3 | 4 | 5 | 6 | 7 |
| 39. Sento una forte scossa elettrica che mi attraversa | 1 | 2 | 3 | 4 | 5 | 6 | 7 |
| 40. Ho bisogno di sentirmi rilassato | 1 | 2 | 3 | 4 | 5 | 6 | 7 |
| 41. Sento un’amplificazione di tutte le emozioni e sensazioni, sia fisiche che mentali | 1 | 2 | 3 | 4 | 5 | 6 | 7 |
| 42. Mi sento protetto dal/la partner | 1 | 2 | 3 | 4 | 5 | 6 | 7 |
| 43. Sento i muscoli delle gambe irrigidirsi | 1 | 2 | 3 | 4 | 5 | 6 | 7 |
| 44. Provo un “senso di vittoria” | 1 | 2 | 3 | 4 | 5 | 6 | 7 |
| 45. Mi sento come se non avessi nessuna preoccupazione | 1 | 2 | 3 | 4 | 5 | 6 | 7 |
| 46. La mia mente è occupata solo dal piacere e dall’oggetto sessuale che lo stimola | 1 | 2 | 3 | 4 | 5 | 6 | 7 |
| 47. Sento una totalità tra mente e corpo | 1 | 2 | 3 | 4 | 5 | 6 | 7 |
| 48. Non penso a nulla | 1 | 2 | 3 | 4 | 5 | 6 | 7 |
| 49. I pensieri che provo in questo momento possono variare di volta in volta | 1 | 2 | 3 | 4 | 5 | 6 | 7 |
| 50. Provo calma | 1 | 2 | 3 | 4 | 5 | 6 | 7 |
| 51. Sento che i miei sensi sono amplificati | 1 | 2 | 3 | 4 | 5 | 6 | 7 |
| 52. Mi sento forte | 1 | 2 | 3 | 4 | 5 | 6 | 7 |
| 53. Provo un piacere quasi trascendentale | 1 | 2 | 3 | 4 | 5 | 6 | 7 |
| 54. Mi concentro esclusivamente sulle sensazioni fisiche e sulle emozioni | 1 | 2 | 3 | 4 | 5 | 6 | 7 |
| 55. Sento dei tremori | 1 | 2 | 3 | 4 | 5 | 6 | 7 |
| 56. Provo una sensazione di conforto | 1 | 2 | 3 | 4 | 5 | 6 | 7 |
| 57. Mi viene da sorridere | 1 | 2 | 3 | 4 | 5 | 6 | 7 |
| 58. In quel preciso momento è puro istinto, senza lasciare molto spazio ai pensieri | 1 | 2 | 3 | 4 | 5 | 6 | 7 |
| 59. Sento che il mio corpo è attraversato da spasmi | 1 | 2 | 3 | 4 | 5 | 6 | 7 |
| 60. Mi focalizzo su quello che succede | 1 | 2 | 3 | 4 | 5 | 6 | 7 |
| 61. Ho la pelle d’oca | 1 | 2 | 3 | 4 | 5 | 6 | 7 |
| 62. Provo esperienze di orgasmo diverse tra loro | 1 | 2 | 3 | 4 | 5 | 6 | 7 |
| 63. Non mi concentro su nulla in particolare | 1 | 2 | 3 | 4 | 5 | 6 | 7 |

The underlined items are different in the masculine and feminine versions.

**OPQ-47: Orgasmic Perception Questionnaire (OPQ; Panzeri, 2016)**

Il seguente questionario contiene delle affermazioni riguardanti l’orgasmo. Legga ogni affermazione e indichi la frequenza con cui Le capita quanto espresso dalla frase, riferendosi esclusivamente alla fase dell'orgasmo e non a quanto accade subito prima o dopo. L’orgasmo può essere provato in diversi modi: risponda sulla base di quello che Lei prova solitamente, riferendosi soprattutto agli ultimi orgasmi provati. Segni con una crocetta la risposta che più si avvicina al Suo modo di pensare e alle sensazioni da Lei provate. Non esistono risposte giuste o sbagliate. Se non ha mai avuto un/a partner, barri pure la casella “non ho mai avuto un/a partner” nelle frasi in cui ci si riferisce esplicitamente ad esso/a.

**ALCUNE DEFINIZIONI**

**Coito**: rapporto sessuale con introduzione del pene in vagina

**Masturbazione**: autostimolazione dei genitali al fine di procurarsi piacere sessuale

**Sesso orale**: rapporto sessuale che consiste nella stimolazione orale (cioè con la bocca) del pene o dei genitali femminili

**Sesso anale**: rapporto sessuale con l’introduzione del pene nell’ano

**Relazione sessuale**: uno o più rapporti sessuali con una o più persone, fini a se stessi che non implichi necessariamente uno scambio affettivo

**Relazione affettiva**: rapporto con uno o più partner che non implichi necessariamente una relazione sessuale

**Rapporto sessuale**: forma di stimolazione reciproca che può comprendere baci, carezze, stimolazione reciproca, coito, sesso orale, sesso anale

| 1 = Mai | 2 = Raramente | 3 = Qualche volta | 4 = Spesso | 5 = Sempre | 6 = Non ho mai avuto un/a partner sessuale | 7 = Non so/ non ricordo |
| --- | --- | --- | --- | --- | --- | --- |

| **1. Provo un senso di potenza** | 1 | 2 | 3 | 4 | 5 | 6 | 7 |
| --- | --- | --- | --- | --- | --- | --- | --- |
| **2. Sento un forte tremolio nei muscoli del corpo** | 1 | 2 | 3 | 4 | 5 | 6 | 7 |
| **3. Mi sento euforico** | 1 | 2 | 3 | 4 | 5 | 6 | 7 |
| **4. Mi sento più sensuale** | 1 | 2 | 3 | 4 | 5 | 6 | 7 |
| **5. Mi sento realizzato** | 1 | 2 | 3 | 4 | 5 | 6 | 7 |
| **6. Percepisco un aumento della temperatura corporea** | 1 | 2 | 3 | 4 | 5 | 6 | 7 |
| **7. Sento una forte tensione di tutto il corpo** | 1 | 2 | 3 | 4 | 5 | 6 | 7 |
| **8. Provo una sensazione di scosse/scariche elettriche che si propagano per il corpo** | 1 | 2 | 3 | 4 | 5 | 6 | 7 |
| **9. Percepisco un piacere fisico localizzato a livello genitale** | 1 | 2 | 3 | 4 | 5 | 6 | 7 |
| **10. Provo un fremito lungo tutto il corpo** | 1 | 2 | 3 | 4 | 5 | 6 | 7 |
| **11. Sento calore sul viso** | 1 | 2 | 3 | 4 | 5 | 6 | 7 |
| **12. Sento delle contrazioni muscolari che si diffondono dall’area genitale al resto del corpo** | 1 | 2 | 3 | 4 | 5 | 6 | 7 |
| **13. Mi sento completamente rilassato a livello muscolare** | 1 | 2 | 3 | 4 | 5 | 6 | 7 |
| **14. Sento una completa incapacità di pensare ad altro** | 1 | 2 | 3 | 4 | 5 | 6 | 7 |
| **15. Ho voglia di urlare** | 1 | 2 | 3 | 4 | 5 | 6 | 7 |

|  | | | | |  | |  |  |  |  | |  |  |  |  |
| --- | --- | --- | --- | --- | --- | --- | --- | --- | --- | --- | --- | --- | --- | --- | --- |
| 1 = Mai | | 2 = Raramente | 3 = Qualche volta | 4 = Spesso | 5 = Sempre | | 6 = Non ho mai avuto un/a partner sessuale | | | | | 7 = Non so/ non ricordo | | | |

| **16. Provo una sensazione di calore nella zona genitale** | | | | | 1 | | 2 | 3 | 4 | 5 | | 6 | 7 |  |  |
| --- | --- | --- | --- | --- | --- | --- | --- | --- | --- | --- | --- | --- | --- | --- | --- |
| **17. Percepisco tensione muscolare** | | | | | 1 | | 2 | 3 | 4 | 5 | | 6 | 7 |  |  |
| **18. Mi sembra che intorno tutto sia perfetto** | | | | | 1 | | 2 | 3 | 4 | 5 | | 6 | 7 |  |  |
| **19. Ho il respiro affannoso** | | | | | 1 | | 2 | 3 | 4 | 5 | | 6 | 7 |  |  |
| **20. Si susseguono in me pensieri positivi riguardo alla realtà** | | | | | 1 | | 2 | 3 | 4 | 5 | | 6 | 7 |  |  |
| **21. Non compaiono pensieri articolati** | | | | | 1 | | 2 | 3 | 4 | 5 | | 6 | 7 |  |  |
| **22. Ho la sensazione di essere isolato dal resto del mondo** | | | | | 1 | | 2 | 3 | 4 | 5 | | 6 | 7 |  |  |
| **23. Vengo investito da un’ondata di piacere che parte dalla zona genitale e si irradia fino alla testa** | | | | | 1 | | 2 | 3 | 4 | 5 | | 6 | 7 |  |  |
| **24. Scompare qualsiasi ansia** | | | | | 1 | | 2 | 3 | 4 | 5 | | 6 | 7 |  |  |
| **25. Non ho alcun tipo di inibizione, di freno o di limite** | | | | | 1 | | 2 | 3 | 4 | 5 | | 6 | 7 |  |  |
| **26. Sento vibrare il corpo come in una scarica elettrica** | | | | | 1 | | 2 | 3 | 4 | 5 | | 6 | 7 |  |  |
| **27. Ho la sensazione che la parte più istintiva di me prenda il sopravvento** | | | | | 1 | | 2 | 3 | 4 | 5 | | 6 | 7 |  |  |
| **28. Mi sento per un attimo fuori dalla realtà** | | | | | 1 | | 2 | 3 | 4 | 5 | | 6 | 7 |  |  |
| **29. Ho la sensazione che il tempo si sia fermato** | | | | | 1 | | 2 | 3 | 4 | 5 | | 6 |  |  |  |
| 1 = Mai | | 2 = Raramente | 3 = Qualche volta | 4 = Spesso | 5 = Sempre | | 6 = Non ho mai avuto un/a partner sessuale | | | | | 7 = Non so/ non ricordo | | | |

| **30. Sento una forte scossa elettrica che mi attraversa** | | | | | 1 | | 2 | 3 | 4 | 5 | | 6 | 7 |  |  |
| --- | --- | --- | --- | --- | --- | --- | --- | --- | --- | --- | --- | --- | --- | --- | --- |
| **31. Ho bisogno di sentirmi rilassato** | | | | | 1 | | 2 | 3 | 4 | 5 | | 6 | 7 |  |  |
| **32. Mi sento protetto dal/la partner** | | | | | 1 | | 2 | 3 | 4 | 5 | | 6 | 7 |  |  |
| **33. Sento i muscoli delle gambe irrigidirsi** | | | | | 1 | | 2 | 3 | 4 | 5 | | 6 | 7 |  |  |
| **34. Mi sento come se non avessi nessuna preoccupazione** | | | | | 1 | | 2 | 3 | 4 | 5 | | 6 | 7 |  |  |
| **35. La mia mente è occupata solo dal piacere e dall’oggetto sessuale che lo stimola** | | | | | 1 | | 2 | 3 | 4 | 5 | | 6 | 7 |  |  |
| **36. Non penso a nulla** | | | | | 1 | | 2 | 3 | 4 | 5 | | 6 | 7 |  |  |
| **37. Provo calma** | | | | | 1 | | 2 | 3 | 4 | 5 | | 6 | 7 |  |  |
| **38. Sento una totalità tra mente e corpo** | | | | | 1 | | 2 | 3 | 4 | 5 | | 6 | 7 |  |  |
| **39. Provo un piacere quasi trascendentale** | | | | | 1 | | 2 | 3 | 4 | 5 | | 6 | 7 |  |  |
| **40. Mi concentro esclusivamente sulle sensazioni fisiche e sulle emozioni** | | | | | 1 | | 2 | 3 | 4 | 5 | | 6 | 7 |  |  |
| **41. Sento dei tremori** | | | | | 1 | | 2 | 3 | 4 | 5 | | 6 | 7 |  |  |
| **42. Provo una sensazione di conforto** | | | | | 1 | | 2 | 3 | 4 | 5 | | 6 | 7 |  |  |
| **43. In quel preciso momento è puro istinto, senza lasciare molto spazio ai pensieri** | | | | | 1 | | 2 | 3 | 4 | 5 | | 6 | 7 |  |  |
| 1 = Mai | | 2 = Raramente | 3 = Qualche volta | 4 = Spesso | 5 = Sempre | | 6 = Non ho mai avuto un/a partner sessuale | | | | | 7 = Non so/ non ricordo | | | |

| **44. Mi focalizzo su quello che succede** | 1 | 2 | 3 | 4 | 5 | 6 | 7 |
| --- | --- | --- | --- | --- | --- | --- | --- |
| **45. Ho la pelle d’oca** | 1 | 2 | 3 | 4 | 5 | 6 | 7 |
| **46. Provo esperienze di orgasmo diverse tra loro** | 1 | 2 | 3 | 4 | 5 | 6 | 7 |
| **47. Non mi concentro su nulla in particolare** | 1 | 2 | 3 | 4 | 5 | 6 | 7 |

The underlined items are different in the masculine and feminine versions.

**ENGLISH TRANSLATION**

**Orgasmic Perception Questionnaire (OPQ; Panzeri, Mauro, Ronconi & Arcos-Romero)**

The following questionnaire contains statements about orgasm. Read each statement and indicate how often you experience what is expressed in the sentence, referring only to the orgasm phase and not to what happens immediately before or after. An orgasm can be experienced in different ways: answer based on what you usually experience, referring especially to the last orgasms you experienced. Mark with a cross the answer that comes closest to your way of thinking and feeling. There are no right or wrong answers. If you have never had a partner, please tick the box "I have never had a partner" in sentences in which you explicitly refer to a partner.

**SOME DEFINITIONS**

**Coitus**: sexual intercourse with the introduction of the penis into the vagina

**Masturbation**: self-stimulation of the genitals to obtain sexual pleasure

**Oral sex**: sexual intercourse consisting of oral stimulation (i.e. with the mouth) of the penis or female genitals

**Anal sex**: sexual intercourse involving the introduction of the penis into the anus

**Sexual relationship**: one or more sexual relationships with one or more people that do not necessarily imply an emotional exchange

**Affectionate relationship**: a relationship with one or more partners that does not necessarily involve a sexual relationship

**Sexual intercourse**: a form of mutual stimulation that may include kissing, caressing, mutual stimulation, coitus, oral sex, anal sex

| 1 = Never | 2 = Rarely | 3 = Sometimes | 4 = Often | 5 = Always | 6 = I have never had a sexual partner | 7 = Don't know/can't remember |
| --- | --- | --- | --- | --- | --- | --- |

1. I feel a sense of power
2. I feel a strong sense of shaking in the muscles of my body
3. I feel euphoric
4. I feel more sensual
5. I feel fulfilled
6. I feel an increase in body temperature
7. I feel a strong tension throughout the whole body
8. I feel a sensation of electric shocks spreading through my body
9. I experience physical pleasure at the genital level
10. I feel a tingle all over my body
11. I feel the heat on my face
12. I feel muscular contractions spreading from the genital area to the rest of the body
13. I feel my muscles completely relaxed
14. I am unable to think about anything else
15. I feel like screaming
16. I have a feeling of warmth in my genital area
17. I feel muscle tension
18. It seems to me that everything around me is perfect
19. I struggle to breathe
20. I experience a flow of positive thoughts regarding reality succeeding one another
21. I don't think complex thoughts
22. I have the feeling of being isolated from the rest of the world
23. I am hit by a wave of pleasure that starts in the genital area and radiates up to the head
24. All anxiety disappears
25. I do not have any kind of inhibition, restraint, or limitation
26. I feel my body vibrating like an electric shock
27. I have the feeling that the more instinctive part of me takes over
28. I feel out of touch with reality for a moment
29. I have the feeling that time has stopped
30. I feel a strong electric shock going throughout my body
31. I need to feel relaxed
32. I feel protected by my partner
33. I feel the muscles in my legs stiffening
34. I feel like I don't have any worries
35. My mind is occupied only by pleasure and the sexual object that stimulates it
36. I don't think about anything
37. I feel calm
38. I feel the strong interconnection between mind and body
39. I feel an almost transcendental pleasure
40. I focus exclusively on physical sensations and emotions
41. I feel tremors
42. I feel a sense of comfort
43. At that precise moment, it is pure instinct, without leaving much because of thoughts
44. I'm focusing on what is happening
45. I have goosebumps
46. I experience different types of orgasms
47. I do not focus on anything in particular
